# Supplementary figures and images for: The impact of monosomies, trisomies and segmental aneuploidies on chromosomal stability
Source: PLoS One. 2022 Jul 1;17(7):e0268579. doi: 10.1371/journal.pone.0268579 (PMC9249180; doi:10.1371/journal.pone.0268579)

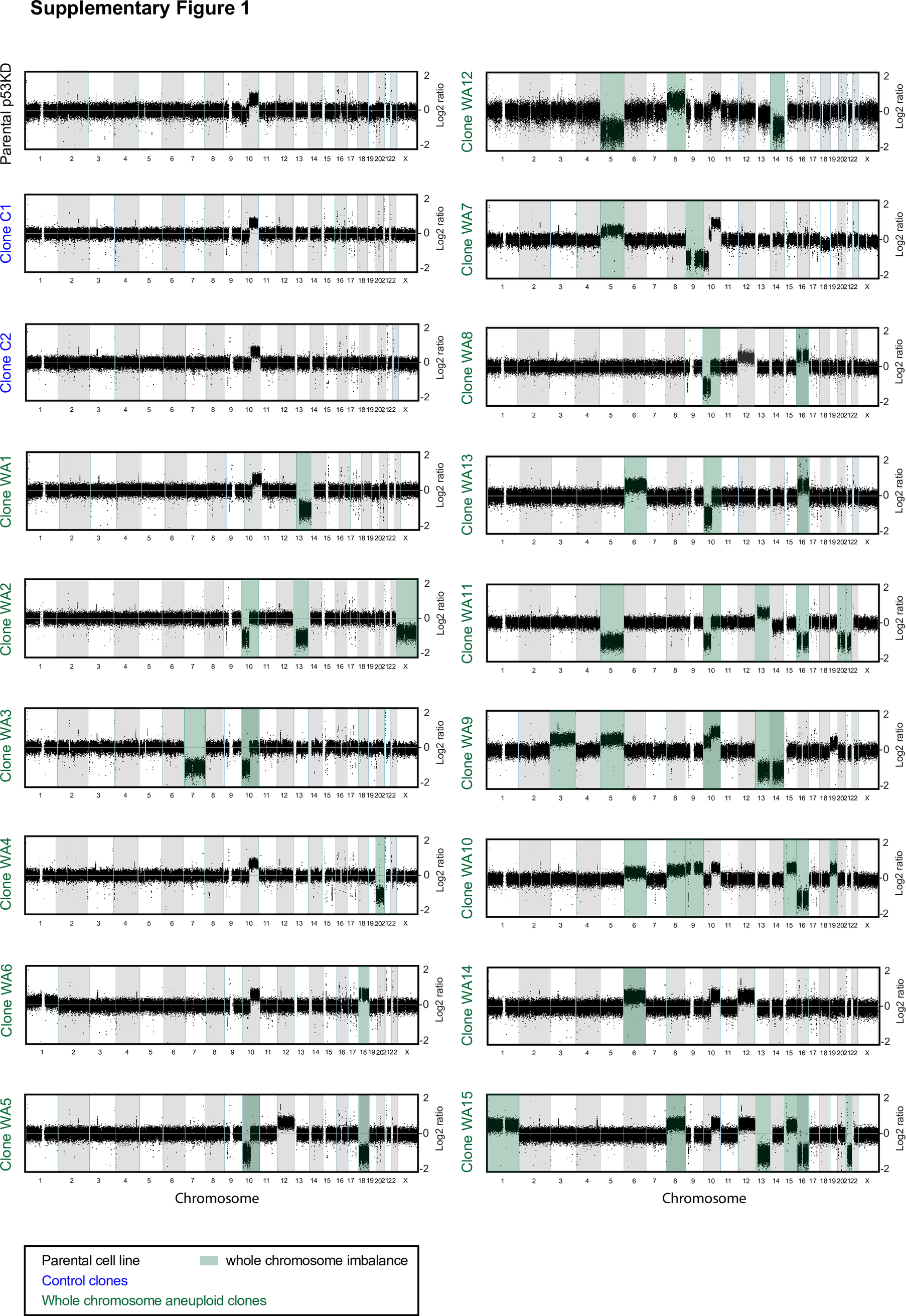

Supplement: S1 Fig — Genome-wide chromosome copy number profile as determined by CNV-seq of the RPE-1 p53kd parental clone (labeled in black), two euploid clones (labeled in blue) and 10 clones harboring solely whole chromosome imbalances (labeled in green). Chromosome gains and losses were depicted in green boxes. Alterations of chromosome 10 and 12, already present in the parental cells, were not highlighted. (TIF) [file pone.0268579.s001.tif]

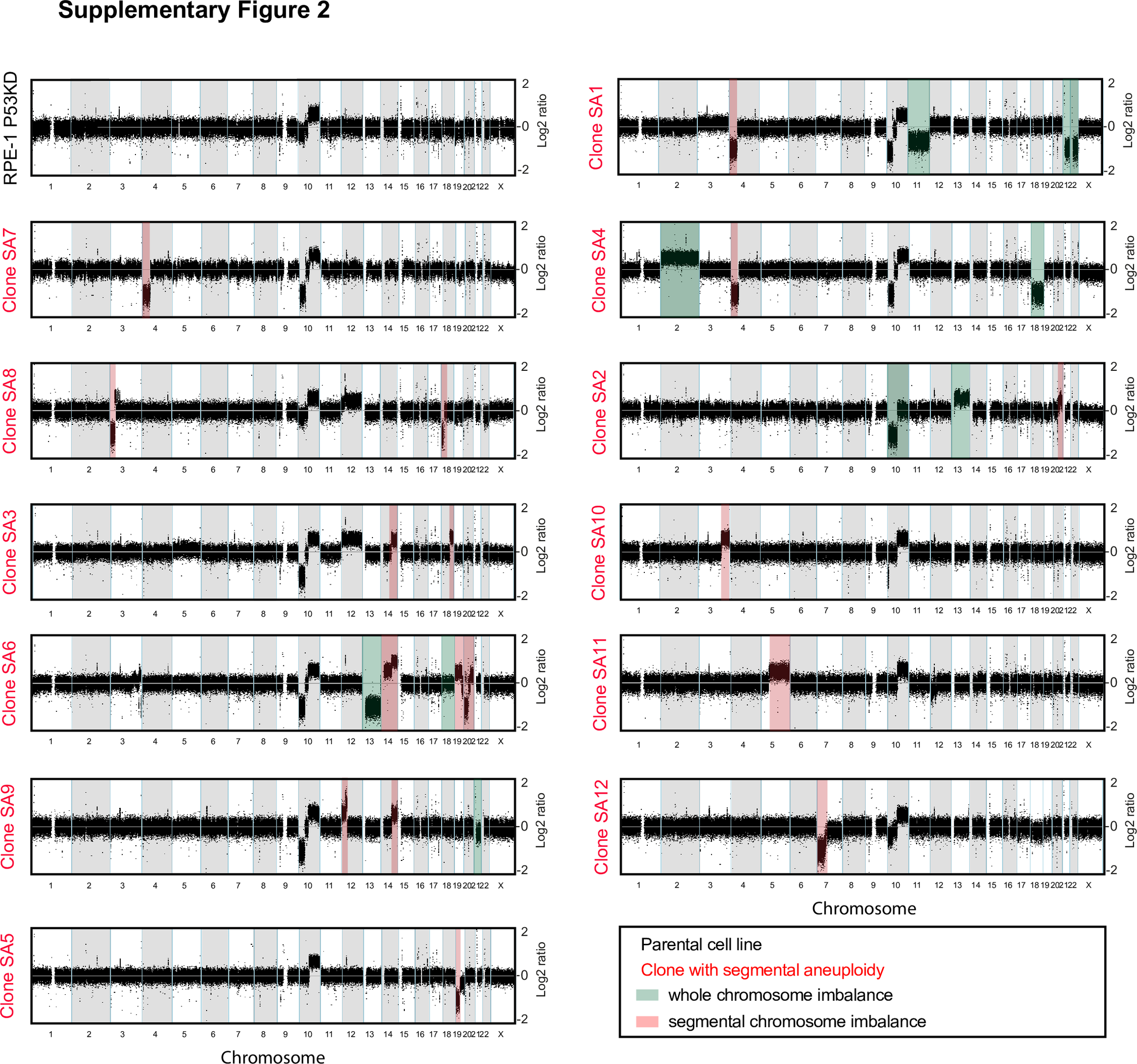

Supplement: S2 Fig — Genome-wide chromosome copy number profile as determined by CNV-seq of the RPE-1 p53kd parental clone (labeled in black), two euploid clones (labeled in blue) and 10 clones harboring segmental chromosome imbalances (labeled in red). Chromosome gains and losses were depicted in green boxes. Alterations of chromosome 10 and 12, already present in the parental cells, were not highlighted. (TIF) [file pone.0268579.s002.tif]

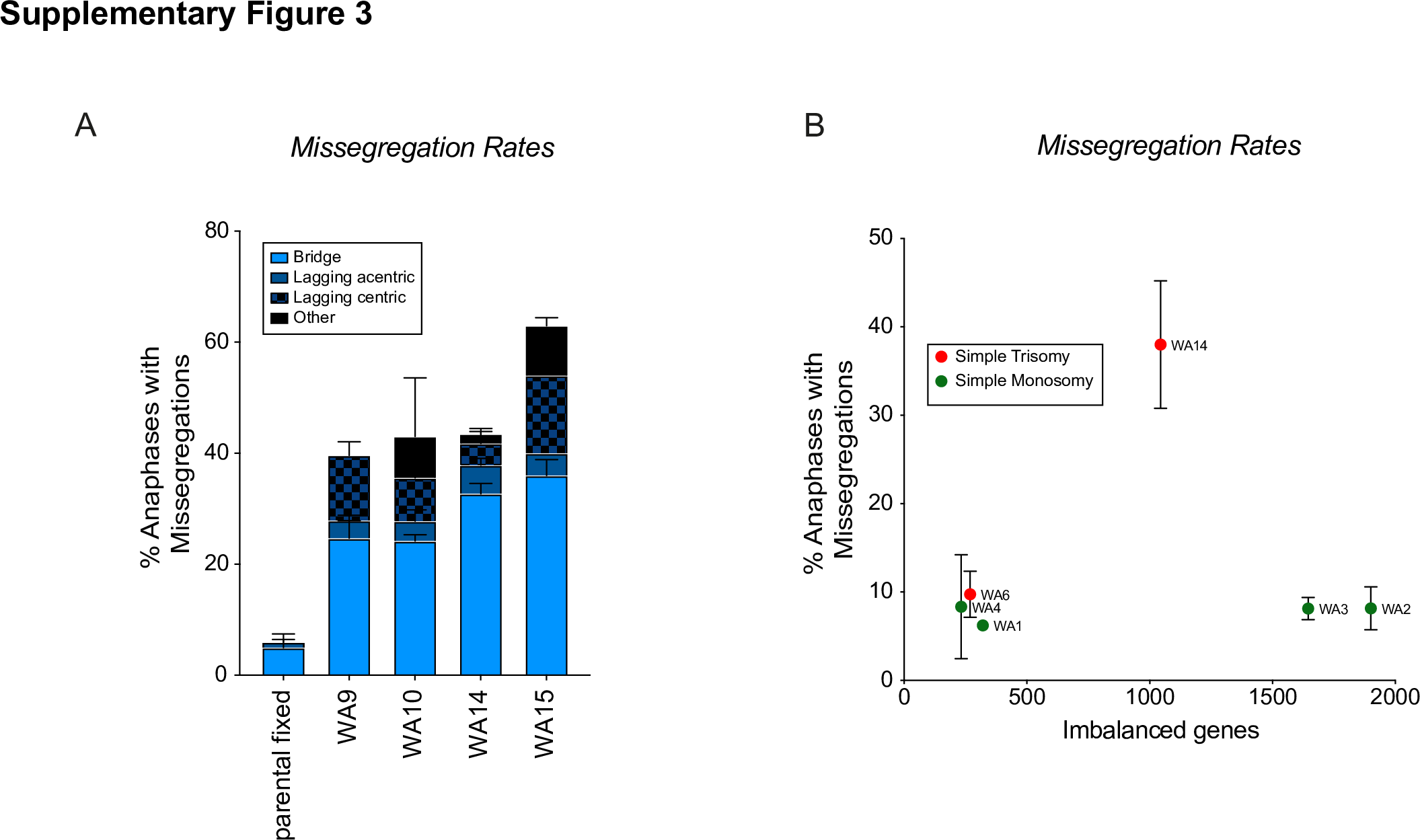

Supplement: S3 Fig — Comparison of simple monosomies and simple trisomies. Simple trisomies show elevated CIN while simple monosomies with comparable levels of gained genes do not. Red dots represent trisomic clones and green dots represent monosomic clones. Missegregation levels as measured in Fig 1B. Error bars indicate standard deviation. (TIF) [file pone.0268579.s003.tif]

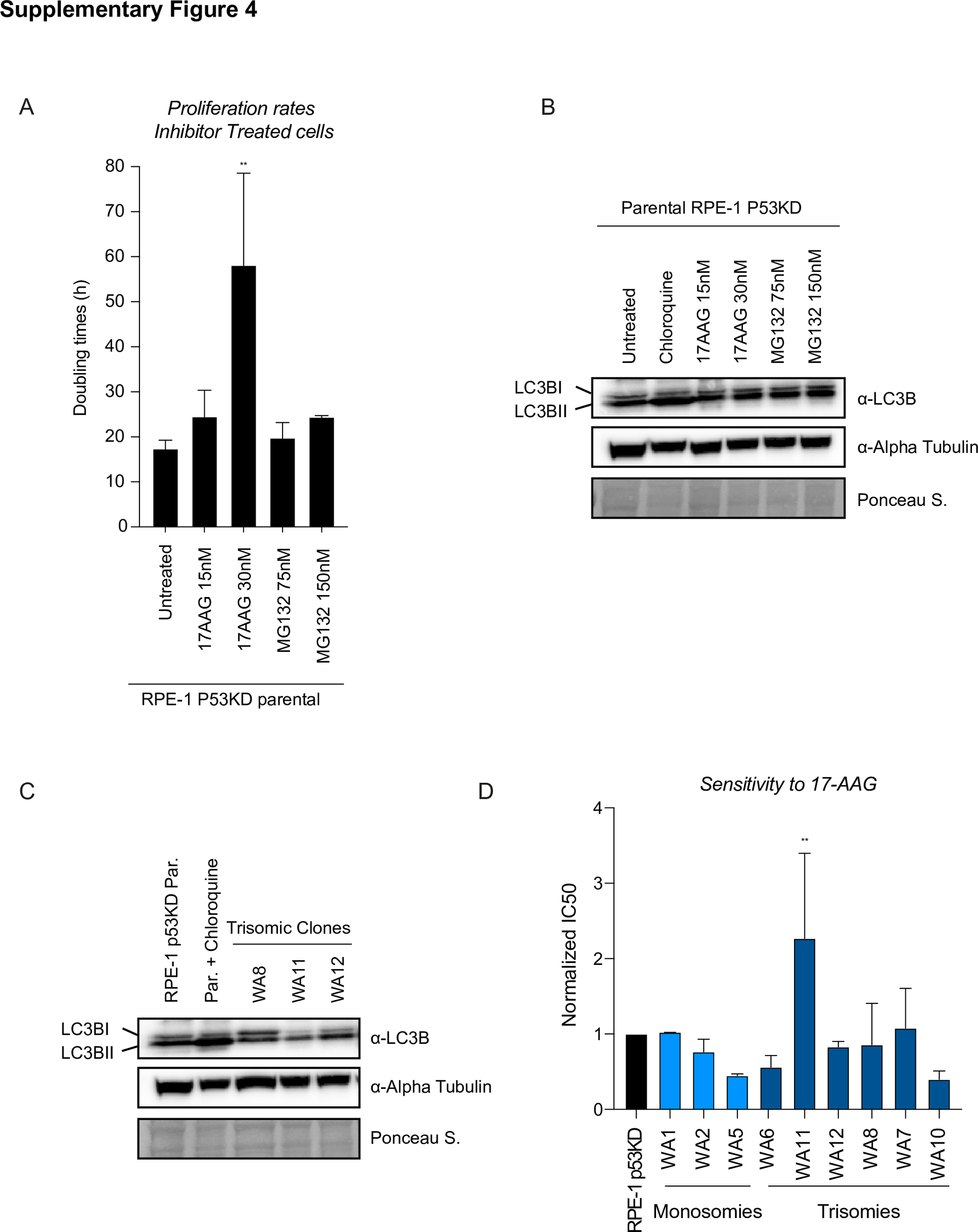

Supplement: S4 Fig — A. Doubling time of parental p53KD cells untreated and treated with different concentrations of proteostasis interfering drugs, as measured in Fig 2. Error bars indicate standard deviation. An ordinary one-way ANOVA was performed between parental and clones. P-values are assigned according to GraphPad standard. B. Immunoblot showing conversion from LC3B-I to LC3B-II in parental p53KD cells untreated, treated with 50uM chloroquine and treated with low doses of proteostasis interfering drugs for 24 hours. Loading control is Alpha Tubulin. C. Immunoblot showing conversion from LC3B-I to LC3B-II in parental p53KD cells untreated, treated with 50uM chloroquine to block autophagy as a positive control and 3 different trisomic clones. Loading control is Alpha Tubulin. D. IC50 values of Hsp90 inhibitor 17-AAG as determined by growth assays of parental p53KD cells and various trisomic clones, ordered per number of gained coding genes. Error bars indicate standard deviation. An ordinary one-way ANOVA was performed between parental and clones. (TIF) [file pone.0268579.s004.tif]

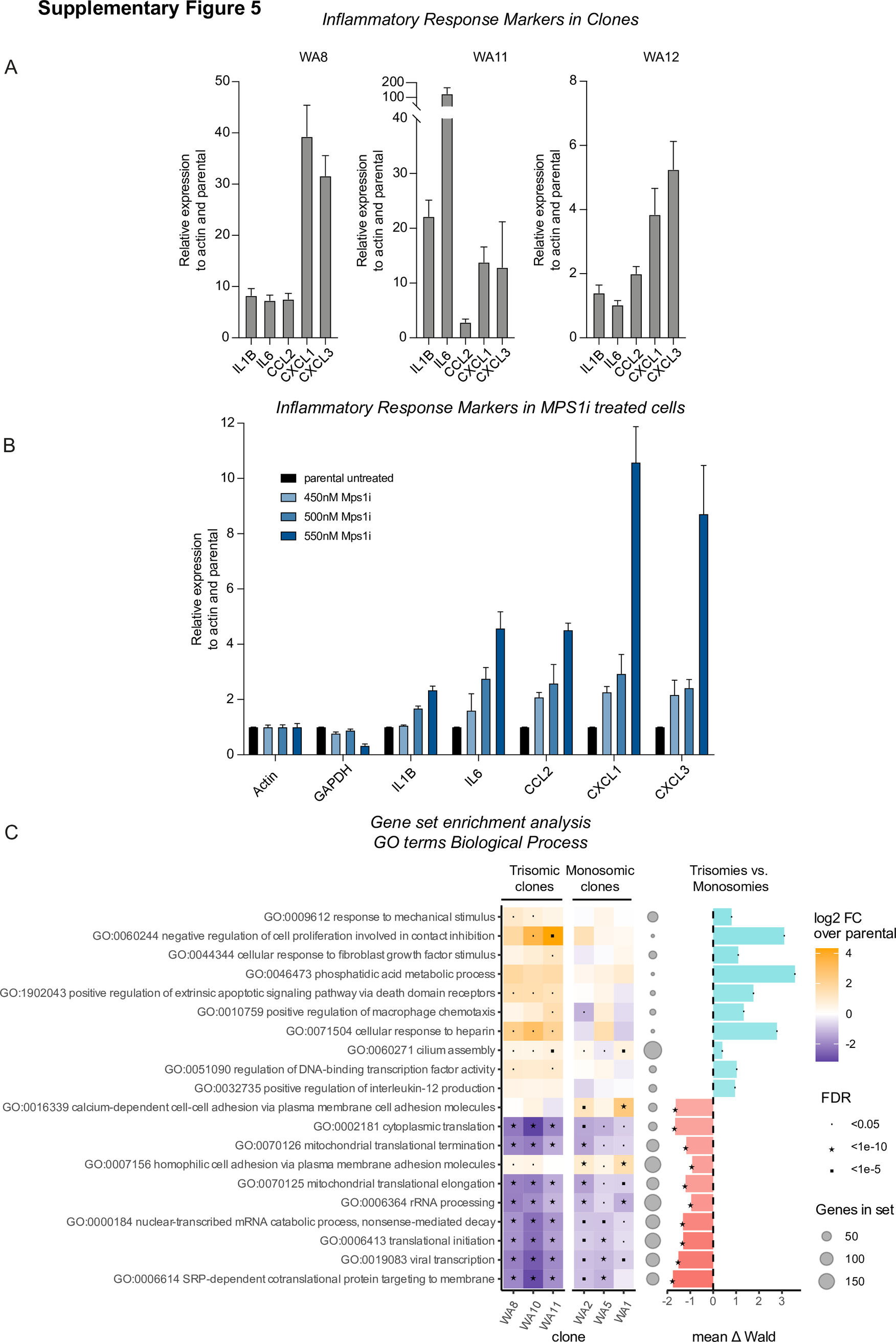

Supplement: S5 Fig — A. mRNA levels of inflammatory response cytokines determined via qRT-PCR in trisomic clones. Values were normalized to Actin and are displayed relative to expression levels in parental cells. Bars show mean expression levels; error bars indicate upper and lower limits. Dashed line represents parental expression levels. B. mRNA levels of inflammatory response cytokines determined via qRT-PCR in parental cells treated with different concentrations of Mps1i for 24 hours. Values were normalized to Actin and relative to expression levels in parental cells. Bars show mean expression levels; error bars indicate upper and lower limits. Dashed line represents parental expression levels. C. Gene set enrichment analysis (GSEA) of RNA sequencing data, evaluating up and downregulated GO Biological Processes in trisomy clones and monosomy clones compared to parental (left graph). Two replicates of every clone were sequenced and the Log2 fold change (FC) was determined compared to parental cells. The false discovery rates (FDR) are indicate with symbols. Largest differences between trisomies and monosomies are shown on the right. Differences in hallmarks between trisomies and monosomies were determined by Wald statistical testing. (TIF) [file pone.0268579.s005.tif]

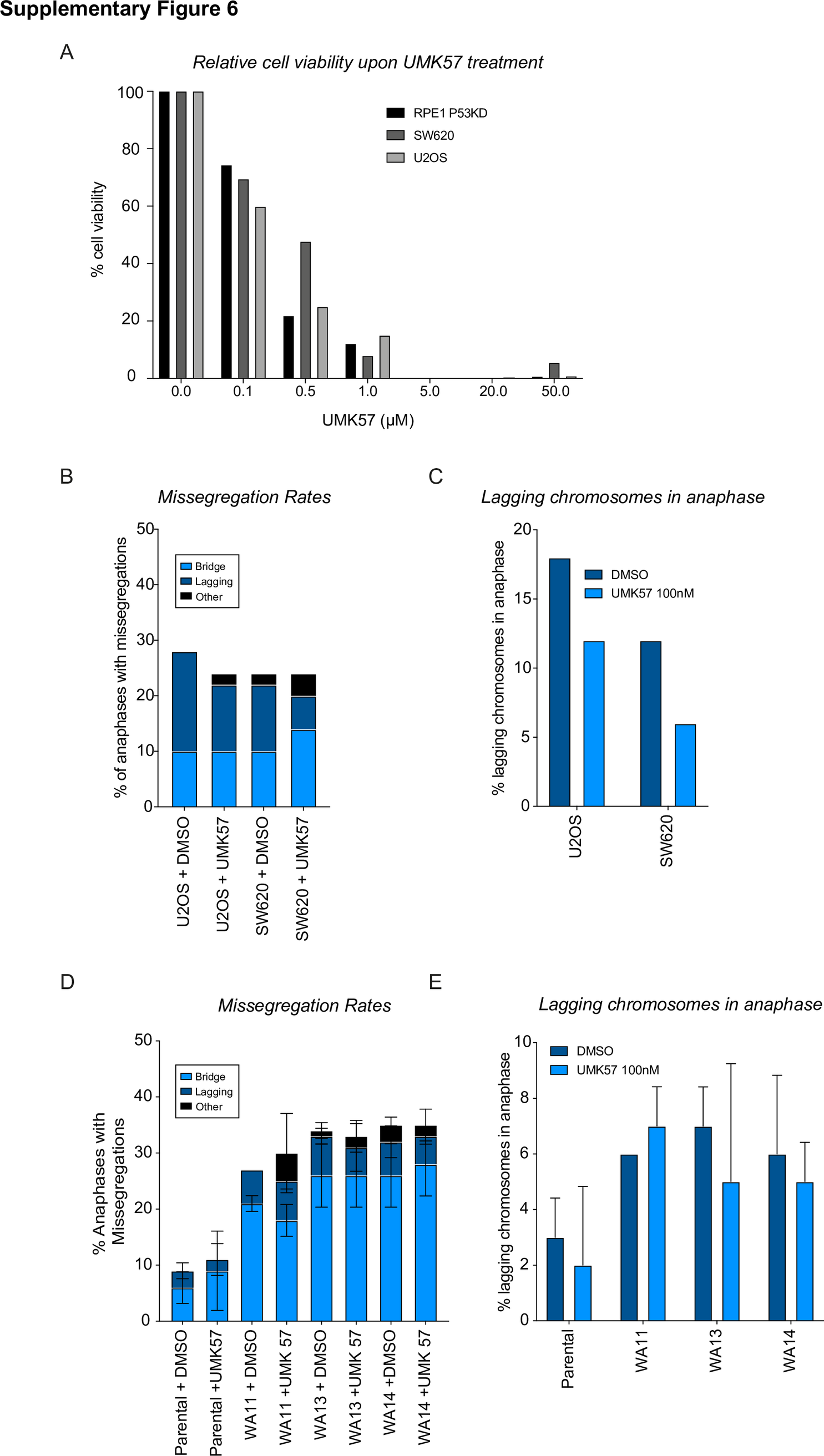

Supplement: S6 Fig — A. Relative cell viability assay in U2OS, SW620 and RPE-1 cells to determine working concentration and compound functionality using Crystal Violet staining. B. Chromosome missegregation rates determined by live cell imaging of U2OS and SW620 cells treated with DMSO or UMK57 (100nM), divided into three subcategories: lagging DNA, anaphase bridges and others (multipolar spindle, polar chromosome, cytokinesis failure, binucleated cell). All conditions were analyzed blinded. Bars are averages of at least 2 experiments and a minimum of 50 cells were filmed per clone. Error bars indicate standard deviation. C. Percentage of only lagging DNA in anaphase in U2OS and SW620 cells. D. Chromosome missegregation rates determined by live cell imaging of RPE-1 parental p53kd cells and 3 different clones treated with DMSO or UMK57 (100nM), divided into three subcategories: lagging DNA, anaphase bridges and others (multipolar spindle, polar chromosome, cytokinesis failure, binucleated cell). All conditions were analyzed blinded. Bars are averages of at least 2 experiments and a minimum of 50 cells were filmed per clone. Error bars indicate standard deviation. E. Percentage of only lagging DNA in anaphase in U2OS and SW620 cells. Error bars indicate standard deviation. T-test analysis revealed no significant changes between DMSO and UMK57 treatment. (TIF) [file pone.0268579.s006.tif]

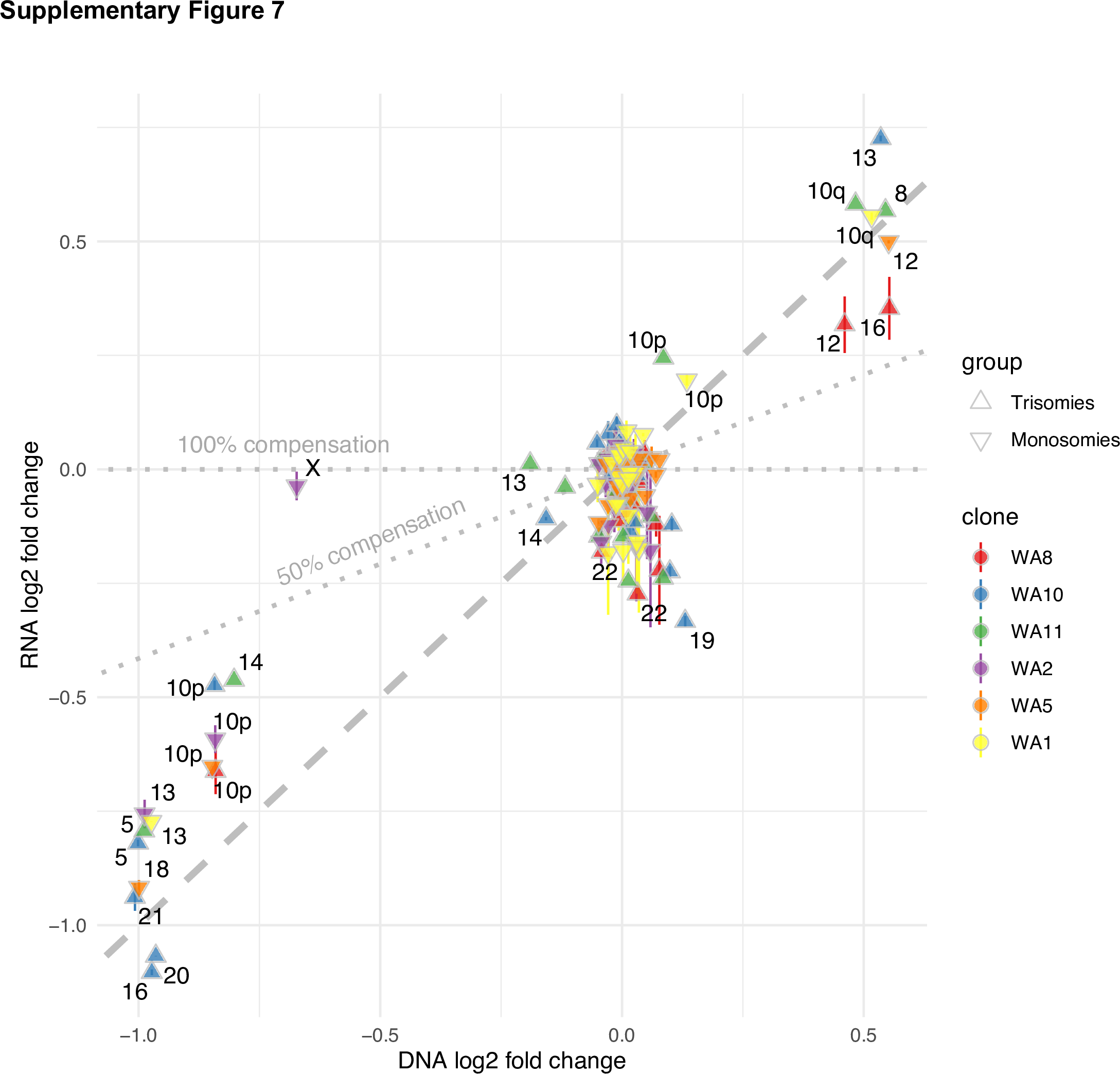

Supplement: S7 Fig — Correlation plotted between gene expression levels and DNA copy number per chromosome. Grey dashed line represents expected value when no compensation is observed. Dotted lines represent 100% compensation or 50% compensation. Upwards pointing arrows indicate trisomy clones, downwards pointing arrows indicate monosomy clones. All chromosomes of each clone are plotted, in representative colors. (TIF) [file pone.0268579.s007.tif]

S1\_raw\_images

Used in Supplementary Figure 4

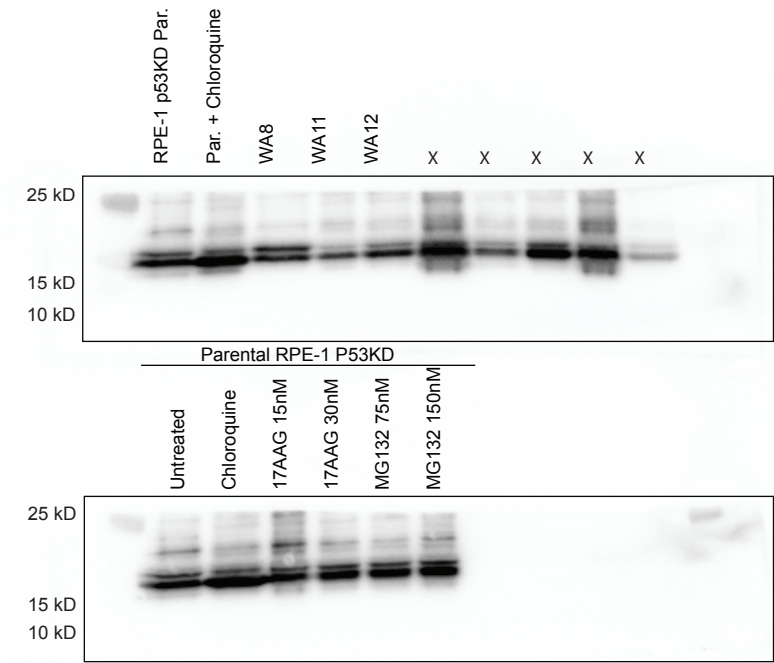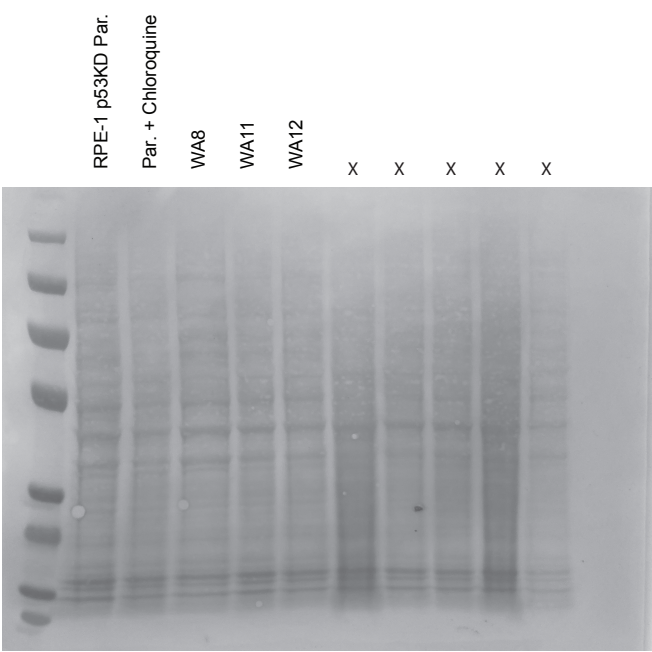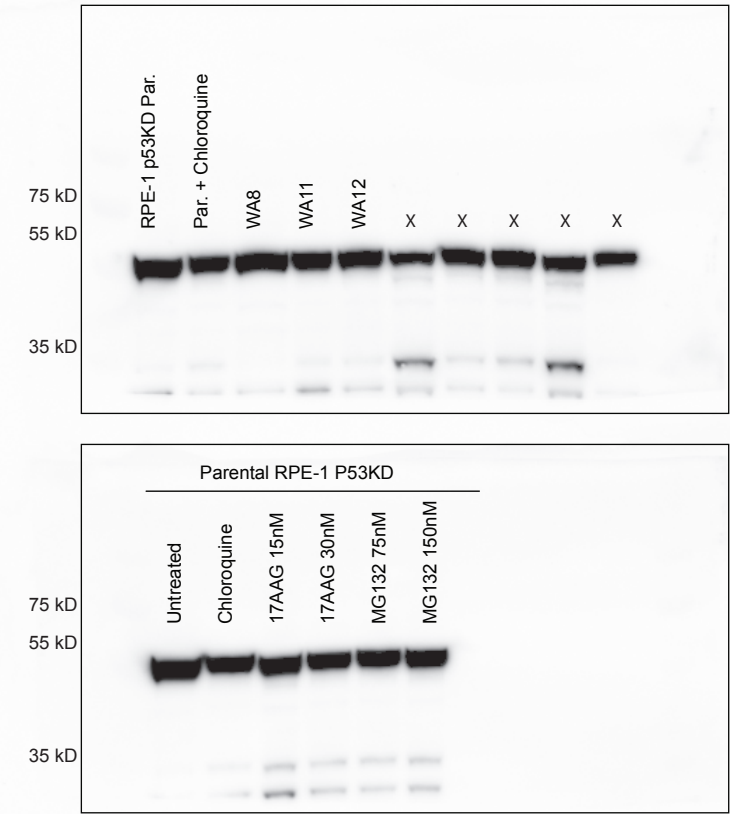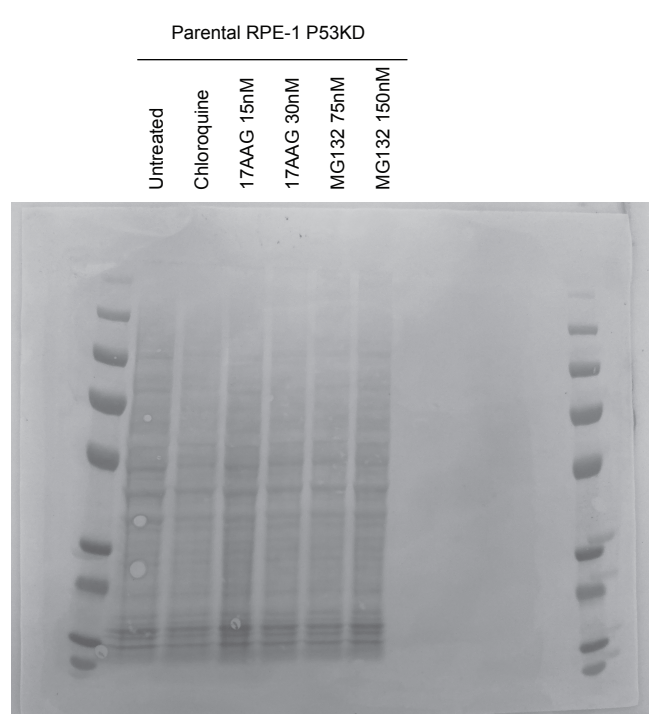

Supplement: S1 Raw images — (PDF) [file pone.0268579.s008.pdf]
