# Supplementary material for: The impact of monosomies, trisomies and segmental aneuploidies on chromosomal stability
Source: PLoS One. 2022 Jul 1;17(7):e0268579. doi: 10.1371/journal.pone.0268579 (PMC9249180; doi:10.1371/journal.pone.0268579)
Supplement: Attachment — Submitted filename: 120776_1_rebuttal_2250828_r49nn4.docx [file pone.0268579.s012.docx]

**Manuscript number:** RC-2021-01073R

**Corresponding author(s):** Jonne Raaijmakers, René Medema

## 1. General Statements

This manuscript provides an observational study of the consequences of different types of aneuploidies on chromosomal stability. We believe that the observations we make can contribute to the field by creating new hypotheses which can be explored in future studies. Furthermore, we touch upon some potential mechanisms that can be at play, which can also be further validated in future work.

## 2. Description of the planned revisions

Both reviewer 1 and 3 suggest to analyze our RNAseq data to investigate potential dosage compensation. We will follow up on these suggestions and add this data to the manuscript. Moreover, reviewer 2 asks if we can attempt to correct CIN using an MCAK agonist. We also will perform this suggested experiment. Please find the point-by-point response to each specific comment below:

**Reviewer 1:**

1. Could the minimal effects of monosomy on chromosomal stability be a consequence of dosage compensation? From the RNA-Seq data that has already been collected, the authors could address whether the transcription of genes on the lost chromosome(s) are upregulated to compensate. This has been suggested in some recent publications (Chunduri and Storchova Nature Comms 2021 and Schukken and Sheltzer bioRxiv 2021).

This is an interesting thought. We will add an analysis of our RNAseq data where we explore if dosage compensation occurs in our clones with whole chromosome aneuploidies to the manuscript. We are planning to investigate this both for trisomic chromosomes as well as monosomic chromosomes to find out if there is any compensation and if there is any difference in the level of compensation between these two classes. This will allow us to compare our findings to the findings of the Chunduri and Schukken studies and we will add these results and discussion to the manuscript.

**Reviewer 3:**

6. The authors perform analyses in which they correlate the number of imbalanced/gained/lost genes with population doubling rates or chromosome missegregation rates. However, as the authors also mention, there may be a certain degree of gene compensation such that not all gained genes are overexpressed and not all lost genes are underexpressed. Since they also performed RNAseq, they could perform a similar analysis by correlating proliferation and chromosome missegregation to the actual number of genes that are over- or under-expressed.

This point is in line with the previous comment of reviewer 1. Indeed, it has been shown by many labs (for trisomies, and two recent papers also for monosomies), that dosage compensation occurs in response to aneuploidy on the level of the proteome. Interestingly, dosage compensation has also been observed on the transcriptome level. As proposed, we will analyze our RNAseq data to see if dosage compensation occurs in our clones and if genes are compensated on a large scale. If we indeed observe dosage compensation, we will calculate the actual gained and lost genes in our clones and adjust our correlation accordingly as suggested by the reviewer.

**Reviewer 2:**

3. The unbiased RNAseq experiments show downregulation of pathways associated with proliferation and with various aspects of metabolism, as expected (and previously shown) for aneuploid cells. The finding that TNFα signaling via NF-κB is strongly activated both upon transient induction of CIN and in trisomic vs. monosomic clones is interesting, but it needs to be validated (e.g., by qPCR and WB of the relevant mRNAs and proteins). The correlation between the transcriptional responses (Fig. 3E) is of interest, but the conclusion that the majority of the differences between trisomic and monosomic clones "can be explained by their CIN phenotype" is over-reaching and not sufficiently supported. In order to back up this claim, it would be necessary to reduce CIN in the trisomic clones (using MCAK overexpression or UMK57) and test whether these transcriptional differences disappear (or at least get attenuated significantly).

The first part of this comment has been addressed (see below). Regarding the second comment on the correction of CIN to validate the claim that the differences between monosomic and trisomic clones are largely explained by their different CIN phenotypes; this is a nice suggestion and we are planning to execute the proposed experiment. For this, we ordered the compound UMK57 and we will treat some of our clones with this compound to see if we can rescue or reduce the CIN levels in these clones. If this is successful, we will determine if the transcriptional differences we find between trisomies and monosomies are no longer present/reduced upon treatment with this compound.

## 3. Description of the revisions that have already been incorporated in the transferred manuscript

Please find below a description of the experiments and comments that were suggested by the reviewers and have already been addressed and incorporated in the manuscript in a point-by-point response.

**Reviewer 1:**

2. Were the microscopy experiments performed in a blinded manner? If so, that can be specified.

The microscopy experiments were indeed analyzed blinded. We now specified this in the methods section.

**Reviewer 2:**

1. The authors found a higher missegregation rates in trisomic clones but not in monosomic clones (Fig. 2). Could this be explained by slower proliferation rates of the monosomic clones? Are the slowly dividing clones shown in Fig. 1A enriched for monosomies? (Coloring the clones based on their trisomy/monosomy status could be helpful.) The correlations between the # of gained/lost genes and proliferation rates (Fig. 2A) do not control for this directly.

This is an interesting thought, however, we in fact observe the opposite: the monosomies are enriched in the faster growing clones (monosomic clones are WA1-5). It needs to be noted that although most monosomic clones grow fast, there is some variation in their proliferation rates: while most indeed proliferate relatively fast (for example clone WA1 and WA3), some proliferate slower (for instance clone WA2 and WA4). Importantly, none of these clones display CIN. As we only start to differentiate between trisomies and monosomies after Figure 2C, we decided not to color code the clones in the plots in Figure 1 to prevent confusion.

2. The proteotoxic stress experiments are preliminary and it is difficult to reach conclusions from the mixed results shown in Fig. 3A and Fig. S3. These preliminary experiments don't really add much to the story, and the manuscript may be better off without them. The same is true for the result shown in Fig. 3B - the statement that trisomic cells activate the PERK-mediated UPR seems somewhat premature given the inconsistent results shown in Fig. 3B.

We think it is important to show what experiments we have done to address the potential presence of proteotoxic stress. It is important to note that the proteotoxic stress pathways are full of negative feedback loops. Hence, an upregulation on RNA level, might not be detected in more downstream signaling cascades. Indeed, consistent with this, we found two phosphatases that are part of a central negative feedback loop in PERK signaling, namely PPP1R15A (also known as GADD34) and PPPR15B (also known as CreP), that are important for the dephosphorylation of eIF2, to be upregulated significantly in our trisomic clones. This might therefore reflect the activation of a negative feedback loop and/or a certain level of adaptation to these proteotoxic stress levels. We have included this data in the revised manuscript and have added this to the discussion of the paper. Because of the significance of the RNA seq data together with the feedback loops that are common in this pathway, we feel that it is warranted to leave this piece of data in as it forms an important foundation for future studies on aneuploidy, CIN and proteotoxic stress. Moreover, a recent study (Xian et al., 2021) finds a strong correlation between PERK-pathway activation and the degree of aneuploidy in a pan-cancer analysis, further supporting our findings. We have now also included this study in our discussion.

3. The unbiased RNAseq experiments show downregulation of pathways associated with proliferation and with various aspects of metabolism, as expected (and previously shown) for aneuploid cells. The finding that TNFα signaling via NF-κB is strongly activated both upon transient induction of CIN and in trisomic vs. monosomic clones is interesting, but it needs to be validated (e.g., by qPCR and WB of the relevant mRNAs and proteins). The correlation between the transcriptional responses (Fig. 3E) is of interest, but the conclusion that the majority of the differences between trisomic and monosomic clones "can be explained by their CIN phenotype" is over-reaching and not sufficiently supported. In order to back up this claim, it would be necessary to reduce CIN in the trisomic clones (using MCAK overexpression or UMK57) and test whether these transcriptional differences disappear (or at least get attenuated significantly).

We have now performed qRT-PCR analysis to support these conclusions and have added this to the manuscript (Fig. S4 A, B). Indeed, we found upregulation of several known NF-κB response genes in both trisomic clones as well as cells with acutely induced CIN.

The fact that CIN induced in parental cells gives a similar response as trisomic CIN cells hints towards a commonality, but indeed a direct causative relationship is not proven yet. To substantiate this claim, we are planning to perform the suggested experiment (correcting CIN using UMK57, also see planned revisions section).

4. The observation of a heterogeneous aneuploidy pattern in clone SA6 (Fig. 5) is anecdotal, and it is therefore premature to infer based on this observation that segmental aneuploidies lead to BFB cycles. In fact, it may be that it is the BFB cycles that led to the observed segmental aneuploidies in this clone to begin with. Concluding from this observation that "monosomies, trisomies and aneuploidies have fundamentally different effects on chromosome instability" (paper's abstract) is a huge leap (also, the results shown in Fig. 4 do not support this claim). This claim should be made only provided that: (a) this phenomenon is shown to recur in clones with segmental aneuploidies; (b) BFB is conclusively ruled out in clones with numerical aneuploidies; and (c) if causality is demonstrated. RNAseq of a few clones with segmental aneuploidies should be performed and compared to the RNAseq of the clones with numerical aneuploidies in order to further explore the potential differences.

We agree with the reviewer that our original title suggested unique effects of segmental and whole chromosome aneuploidies on CIN and our choice of words suggested that BFB-cycles are a phenomenon occurring solely in segmental aneuploidies. However, we agree that this phenomena is likely not limited to segmental aneuploidies. In fact, chromatin bridges are observed regularly in clones harboring whole chromosome aneuploidies as well, and these bridges can result in broken chromosomes and thus instigate a BFB-cycle as well. Since we have a limited number of clones we could only prove that one clone experienced BFB-cycles, making this finding indeed anecdotal. We tried to find evidence for these cycles in other clones but did not find proof for their presence.

To clarify that BFB-cycles are not limited to segmental aneuploidies and to acknowledge that segmental aneuploidies and whole chromosome aneuploidies actually mostly behave the same in terms of their CIN phenotypes we decided to 1. adjust the title of the manuscript to: “The impact of monosomies, trisomies and segmental aneuploidies on chromosomal stability”. And 2. to adjust the results and discussion sections to emphasize that we found evidence for a BFB-cycle in one of our clones with a segmental aneuploidy but that is likely not exclusive to such clones.

5. A recent study from the Sheltzer group (Schukken and Sheltzer; bioRxiv 2021) compared protein dosage compensation in aneuploid human cancer cell lines, and looked specifically into potential differences between gains and losses. A comparison of the results of the current study to that of the cancer cell line study would be of interest.

We agree that comparing our findings with the paper published by Jason Sheltzer is important and we added a section to our text where we elaborate on this. They made some very interesting findings that could have links to our study. First, they show that chromosome gains and losses both perform dosage compensation to similar extents and second, they speculate that dosage compensation on gained chromosomes and lost chromosomes occurs via different mechanisms. In the previous version, we already speculated on a similar mechanism but we have now implemented and discussed the study of Schukken et al. in this perspective.

****Minor points:****

1. How were the data processed from DNA sequencing to CNA calling? The generation of the plots shown in Fig. S1 and S2 is described neither in the Figure Legends nor in the Methods.

This was indeed not described in the previous version. We have now added it to the methods section.

2. Whenever a conditional Spearman test is performed, it should be explicitly mentioned which variables X and Y were compared and what was the covariate Z (it's not immediately obvious for some of the analyses).
   We agree with the reviewer that it was unclear and added the variables which were compared in the text.

**Reviewer 3**

1. The authors argue that previous studies (e.g., Nicholson et al., 2015 and Passerini et al., 2016) had examined small numbers of aneuploid cell lines and that the cell lines analyzed in those studies only carried trisomies, but not monosomies. Here, instead, the authors generated a large number of cell lines, with both trisomies and monosomies. However, most of the cell lines that the authors define as trisomic are actually characterized by complex karyotypes with both trisomies and monosomies. This makes the interpretation of the data not very straightforward and raises the question as to whether it is fair to lump all these cell lines together in a "trisomy" category. In my opinion, it would have been better to sort the cell lines in more groups and specifically, simple monosomies, simple trisomies, and complex karyotypes.

We agree with the reviewer that a subset of our trisomy clones have more complex karyotypes. To simplify, we now added an additional analysis, only focusing on the simple karyotypes, as suggested by the reviewer. Here, we compared simple trisomies (harboring one or two gains with no additional aneuploidies) with simple monosomies (harboring one or two losses with no additional aneuploidies) and we still observed major differences between trisomies and monosomies. Most strikingly, trisomy clone WA14 has a larger number of gained genes as compared to the number of lost genes in WA2 and WA3 but WA14 has extremely high CIN levels, whereas WA2 and WA3 did not show CIN (Fig. S3). This further underlines our hypothesis that extra genetic material drives CIN while we did not find evidence that loss of genetic material does so.

2. The authors present the monosomic cell lines as a novelty of their study and I agree with this statement. However, their data show that monosomy does not have an effect on CIN, thus dampening the significance of this part of the study.

We understand that it seems counterintuitive that monosomies that do not show CIN could help us to understand CIN. However, the ‘negative’ results regarding monosomies definitely make a critical contribution to our understanding of the aspects driving CIN in response to aneuploidy. We found that losing genes does not instigate a CIN phenotype and this finding was not only based on the monosomic clones but also on the clones harboring a combination of gained and lost genes. Therefore, these data are extremely relevant for our understanding of what aspects of aneuploidy instigate CIN. In this case, negative data can be of value and in our opinion contributes to the significance of the study.

3. The other finding the authors present as particularly relevant is evidence that an initial chromosome breakage led to B-F-B cycles in clone SA6. However, the emphasis placed on this observation is unwarranted for several reasons. First, this observation is far from novel given that the B-F-B cycle as a consequence of chromosome breakage was discovered by Barbara McClintock over 80 years ago. Second, chromosome bridges are as common in the SA clones as they are in the WA clones and B-F-B cycles may also be going on to some extent in the WA clones (e.g., as a consequence of micronucleus formation). In fact, the authors focused specifically on clone SA6, in which the segmental imbalance in chromosome 14 seems to occur concomitantly with an increase in copy number for the same chromosome. The authors describe the point on possible bridge formation in WA clones in the discussion section on page 14, but they say that it may be possible that bridge formation also occurs in the trisomic clones and completely disregard the fact that in a number of their WA clones the increase in chromosome missegregation appears to be driven by an increase in chromosome bridges. Thus, it is a fact (and not a possibility) that chromosome bridges are occurring in the trisomic clones and in some cases at rates that are similar or higher (e.g., WA15) than in the SA clones.

Please also see our reply to reviewer 2 point 4. We agree with the reviewer that chromatin bridges are indeed present both in WA clones and SA clones, and overall to similar extents. We chose to make a distinction between WA and SA clones as SA clones initially experience DNA damage due to chromosome breakage upon their generation, which could require some level of adaptation to tolerate this damage and this could impact chromosomal instability and we now clarify this better in the text. However, we indeed observed that WA and SA clones have similar CIN patterns that are largely explained by the number of gained genes. We have now adjusted the title and made several important adjustments to the text to clarify that the observation of the BFB-cycle in this single SA clone is anecdotal and likely not limited to segmental aneuploid clones but is likely to also occur in WA clones.

5. The other type of experiment that I had concerns about was the population doubling experiment, which appears to have been performed with technical replicates, but no biological replicates (i.e., repeated only once). I believe the authors must show reproducibility by repeating the experiment multiple times, on different days.

We have now added biological replicates to the results. The doubling times were comparable. We updated the graph with the proliferation rates of each clone, added error bars, and updated our correlation analyses.

****Minor points:****

1. The details of the treatment used to induce the initial chromosome missegregation is not described anywhere. The authors refer to a previous publication. However, I think the details of the treatment should be included in the methods section of this manuscript.

We have elaborated more on the generation of the aneuploid clones in our methods section of the manuscript.

2. Statistics should be added to all the bar graphs.

We have now performed the appropriate statistics and added this data to the graphs.

3. The error bars in some of the chromosome missegregation graphs are not aligned correctly. I cannot envision how this happened, but it must be corrected.

This must have happened during moving the graph in illustrator. We apologize for not noticing this and we have now aligned the error bars appropriately.

4. In panels A and B of figure 1, it would be important to display the data for the various clones in the same order to allow the reader to more easily compare the data reported in the two graphs.

We agree with the reviewer that this will make it easier to compare the data between the two graphs and we have updated the graphs accordingly.

5. In figure 1C and all other scatter plots included in the manuscript, the individual dots should be labeled with the name of the corresponding clone. If the dots appear too close together for labeling, they could be spaced out by increasing the width of the graph (i.e., stretching the graph out width-wise) and/or introducing a break in the X-axis.

We have now labeled the dots with their respective clone name.

6. I was surprised to find that 3% of the control cells had micronuclei. Micronuclei in RPE-1 cells with wt p53 occur at rates that are one order of magnitude lower.

This number is in fact in line with a recent study in RPE1 P53KO cells performed by the Storchova lab (~2,5%, Chunduri et al. 2021) and also reflects what we found before (~2%, Soto et al. 2017). Importantly, loss of p53 can lead to enhanced missegregations under normal conditions (Drost et al. 2015, Soto et al. 2017), which might contribute to these relatively high levels of MN.

7. The authors should provide information about what the different colors in Figure 3E mean.

We agree and we have added this to the figure legends.

8. Figs. S3A and S3C. One of the labels uses both the Greek symbol for alpha and the word "alfa."

We agree with the reviewer that this is unclear. We have changed the Greek symbol alpha, referring to the antibody, to anti. We realize the spelling mistake in alfa and will change it to the correct alpha-tubulin.

9. Fig. S3D. The authors only brush over the data here. However, it is noticeable that there is a large difference between clone WA11 and clone WA10. Importantly, these two clones differ in the types of aneuploidies they carry. Specifically, WA 11 mostly carries monosomies, whereas WA10 mostly carries trisomies. The authors may want to discuss their observation in light of the karyotypic differences between these two clones.

Indeed, WA10 and 11 have important differences as fairly pointed out by the reviewer. We mention in the text that WA10 is the clone with the most gained genes and therefore it makes sense that it is the most sensitive to 17AAG. WA11 is the clone that carries mostly monosomies but it also has a trisomy and as we argue that gaining genes drives instability and proteotoxic stress, thus we expected that this clone would be more sensitive to the compound compared to parental cells, as it also showed increased instability. As this was not the case, it was indeed possible that it rather was the total number of imbalanced genes that influences the sensitivity/resistance to the compound. To test this, we now correlated both the number of imbalanced genes as well as gained genes to the sensitivity of the compound but both correlations were not significant.

10. Fig. 5E. Imaging information for this experiment was not provided.

We apologize for the lack of information and we have now added these details to the manuscript.
11. Top of page 11, end of results section. The authors state "We hypothesize....." What the authors are doing here is offering an interpretation of their data, so "hypothesize" may not be the best word choice. They may want to consider rephrasing.

We agree and rephrased it to “might suggest”.

12. Discussion section titled "Monosomies are chromosomally stable." Some parts of this section are a bit confusing and overall, the section seems to lack focus. The authors summarize data that are not related to the monosomic clones, but they do not provide a detailed discussion of how their data compare to the data by Chunduri et al. For instance, one of the monosomic lines generated here carries the same monosomy 13 that Chunduri et al. described in both of their genetics backgrounds. It would be helpful to have some focused discussion on this specific comparison rather than the very general discussion that is currently in this section.

We agree with the reviewer that a specific comparison is warranted. In fact, the clone 13 in the p53kd background used in the study by Chunduri was a gift from us to their lab. In agreement with our findings, they do not observe CIN in this clone, only when chromosome 13 is lost in a p53KO background. However, this specific clone carries a segmental gain of chromosome 22 in the background, that might explain the enhanced CIN levels. We now elaborate in more detail to provide explanations for the apparent discrepancies between the study of Chunduri et al. and our own data. We also have re-organized this section to make it more focused.

13. Overall, the manuscript is well written, but there are a few minor issues with wording and punctuation.

We have double checked our wording and punctuation and have made several adjustments.
14. The figure legends could benefit from the addition of some more details.

We have added extra details to the figure legends where needed.

15. Some references appear to be missing from the reference list. For instance, I noticed that Kojima and Cimini, 2019 is cited in the text, but does not appear in the reference list. I just happened to notice this one, but there may be others.

We apologize for this and we have now checked and corrected the reference list.

## 4. Description of analyses that authors prefer not to carry out

**Reviewer 2:**

3. Can any of the findings be generalized beyond the specific system of p53-inactivated RPE1 cells? 100% of the results shown in the paper are based on this one cell line, which is aneuploid to begin with (with what seems to be a clonal gain of chromosome 10q and a subclonal gain of chromosome 12). To examine the generalizability of the identified correlations (e.g., the association between CIN and gains but not losses), they should be tested in human tumors, cancer cell lines, and/or additional non-transformed cellular systems. Of particular note in this context is the fact that, as appropriately mentioned in the Discussion, a recent study from the Storchova lab (Chunduri et al. bioRxiv 2021) found that some monosomies do induce CIN, calling into question the general conclusion of the current paper that there's a fundamental difference between trisomies and monosomies in this regard.

We completely agree that adding other cell lines would contribute to generalizing our findings. However, finding a second immortalized, non-aneuploid cell line has proven to be challenging. We already investigated BJ, MCF10A and HFL cells but their proliferation rates were extremely slow to start with and inducing aneuploidy would exaggerate that even further, making these cell lines difficult to work with in context of aneuploidy research. As a second system we therefore aimed to generate aneuploid clones in the fast proliferating HCT116 cancer cell line that has a near-diploid karyotype and is chromosomally stable. However, we experienced high rates of tetraploidization (both spontaneous as induced by our treatments), which would interfere with the interpretation of our results. Taken together, thus far we have failed in identifying an alternative cell line suitable for generating additional clones. Even if we now invest time in finding an additional cell line, and we would succeed, it needs to be noted that the process of generating aneuploid clones is extremely time consuming. It requires the manual scanning of 1000^th^ of single wells to identify wells containing single cells to make sure that we were dealing with a clonal population. Growing these single cells to obtain full clones required minimal 4 weeks but usually longer as aneuploidy interferes with proliferation rates in most cases. After obtaining clones we performed DNA sequencing to find out which clones had an aneuploid karyotype, which usually takes >12 weeks in our in-house facility. The overall success rate of this approach was ~ 17%. Therefore, we believe that for this study, investing in a new cell line (with the risk that we won’t succeed) will take up too much time.

Taken together, we see this study as a nice foundation for future studies where these clones or the method for generating these clones can be used. Importantly, the obtained data will contribute to the progression of the field and therefore we feel that publishing it is warranted. Nevertheless, we do believe for our future studies generating new clones in a different genetic background is valuable and it is something that we are investing in.

4. I also have concerns about some of the experimental approaches/designs. For instance, all the missegregation data were derived from live cell imaging experiments that were performed at low magnification and with fluorescent labeling only for the DNA. Yet, the authors classify some of the errors as lagging chromosomes. I think it is objectively impossible to discriminate between a lagging chromosome and a chromosome fragment at low magnification without a kinetochore marker. Moreover, despite the imaging being done at low magnification (which should allow easy data collection), the experiment was repeated only two times and only 50 cells per experiment were analyzed. Finally, it is not clear what the category "other" refers to. To a minimum, these results should be supported/confirmed by an analysis of chromosome segregation defects in fixed cells stained for DNA, kinetochore, and microtubules. This type of analysis could be performed at higher resolution, allowing unambiguous classification of different types of errors. Moreover, hundreds of cells per sample could easily be analyzed.

The reviewer expresses some concerns regarding the experimental approaches mostly related to our missegregation analysis. We agree with the reviewer that immunofluorescence experiments enable more detailed analysis of the type of missegregation. In this study we chose for a more observational approach where we focus on broader categories of missegregations (lagging, bridges, multipolar anaphases, binucleation) which can be defined reliably by live cell imaging. We will respond to each concern separately:

1. Low magnification: The microscope setup that we used has a pixel size of 0,6um per pixel at the applied settings (20x, 2x binning). As the size of chromosome varies between 1-20 uM this resolution is high enough to see individual lagging chromosomes with high certainty. We added some example images to illustrate this.

2. Labeling DNA only: For our missegregation categories we do not distinguish between entire lagging chromosomes and lagging chromosome fragments, so for our purpose it is not necessary to use a kinetochore marker.
3. Categories: We agree with the reviewer that the category “others” was not well defined and we have elaborated more on it in the text.
4. Applying fixed analysis: The purpose of this study was to investigate levels of missegregations across a whole panel of aneuploid clones to see if the degree of aneuploidy correlates with missegregation rates. We agree with the reviewer that fixed imaging could provide more information about the exact type of errors but for this study the analysis we performed currently and the classifications of the errors are sufficient to establish instability levels per clone and generate correlations.
5. Low number of cells analyzed: It needs to be noted that a lot of cell lines that we imaged have extremely low proliferation rates. Thus, both for fixed and live cell analysis it is quite challenging to obtain enough anaphase cells to analyze. The suggestion to switch to fixed analysis for quantification will likely not solve this issue. Missegregations can only reliably be scored in a short phase of anaphase, when chromosomes are pulled apart far enough but not decondensed yet. This might be a time window of ~5-10 min. Thus, in a cell line with a doubling time of 30 hours, this means only ~1:360 cells will be in this phase at any fixed time point. To be able to score ~200 cells it means 36000-72000 cells need to be checked in order to find enough anaphase cells (and this adds up in clones with even slower proliferation rates). With live cell imaging you at least capture every cell going through mitosis.

Taken together, we feel that a fixed experiment will not easily enhance the number of cells analyzed. Also, in the current study we did not make a distinction between lagging fragment or full chromosomes so the added benefit of this data is unclear at this moment. We will specify in the text that the category lagging chromosome includes whole chromosomes and chromosome fragments for clarity but we feel that adding the fixed analysis is extremely time consuming with limited to no added benefit.
