# Supplementary material for: The impact of monosomies, trisomies and segmental aneuploidies on chromosomal stability
Source: PLoS One. 2022 Jul 1;17(7):e0268579. doi: 10.1371/journal.pone.0268579 (PMC9249180; doi:10.1371/journal.pone.0268579)
Supplement: Attachment — Submitted filename: Response to reviewers.docx [file pone.0268579.s014.docx]

**Response to reviewers**

**Reviewer #2:**

The authors made some effort but were unable to successfully address the two issues that were highlighted by myself and by Reviewer #3 in the previous round of comments:
(1) The dosage compensation analysis that is shown in the Discussion and in Supplementary Fig. 7 is partial and incomplete. A pathway-level compensation analysis should be performed, and so is a more direct comparison to the published literature on that matter.

In the preprint by Schukken et al., they investigated dosage compensation both on RNA level as well as protein level using a very large data set containing 367 cell lines from the Cancer Cell Line Encyclopedia (CCLE). They found that mean mRNA and protein expression levels increases upon arm gain and decreases upon arm loss. However, on the individual gene level, they observed that a large subset of genes was ‘buffered’. However, it is very important to note that this observation mostly applied to buffering at the protein level as the majority of RNA transcripts scaled with the DNA copy number (in line with previous studies, see Fehrmann et al. 2015). The Schukken study performed GO and pathway enrichment for the genes that were found to be buffered. When performing this analysis for proteins, they found enrichment for ribosomal genes, RNA processing genes and protein complex genes. These findings nicely reflect previously published results for chromosome gains (Stingele et al. 2012, Dephoure et al. 2014, Brennan et al. 2019). Importantly, Schukken et al. found similar Gene Ontologies and pathways to be buffered for gains and losses. A rather different result was obtained when performing a similar analysis for buffered RNA’s: first, they only found three pathways to be enriched, which were only marginally significant. Second, these pathways were different between gains and losses and did not seem to involve expected pathways. Therefore, the authors concluded that: “*This lack of dosage compensation at the RNA level indicates that translational or post-translational regulation, rather than transcriptional regulation, drives the pervasive dosage compensation of buffered proteins and protein complex subunits that we have observed in aneuploid cancer cells*.”. We completely agree with this line of reasoning and therefore, we feel that performing pathway enrichment on our transcriptome data would not lead to any relevant insight.

In summary, although we could try to perform pathway enrichment analysis on our data set, we believe this will not result in meaningful data for the following reasons:

1. Our data set is way too small to reliably assign genes to be buffered or not. RNA sequencing data is intrinsically noisy and for most aneuploidies we only have one clone with that specific aneuploidy. Therefore, we are not able to reliably classify a gene as buffered or not with sufficient confidence.
2. Most buffering occurs on protein level, as also found by Schukken et al. and as suggested by many other publications such as by Gonçalves et al. 2017. Since we only obtained RNA data, these findings will unlikely result in any valuable conclusions.

Thus, if we would do such analysis, it would at most lead to insignificant data that rather than leading to pushing the field forward, it could lead to confusion in the field. To address the questions regarding dosage compensation on transcriptome level on the individual gene level/pathway level, a much larger data set is required, preferentially covering all chromosomes multiple times. Instead, we propose to highlight the limitations of our data set in the manuscript and make clear that larger datasets are required to make solid conclusions on compensatory mechanisms on the transcriptome and proteome level. We discussed this with the scientific editor and we agreed to clarify these limitations in the discussion.

In the discussion we state the following:

*“However, we need to note that the amount of data is limited and that there is also noise observed around the chromosomes that are euploid. Also, we lack the resolution to conclude if there is dosage compensation on the level of individual, possibly critical, genes. Finally, and most importantly, to make solid conclusions on dosage compensation, we require proteome data as we assume the biggest consequences of dosage compensation come from mechanisms associated with protein degradation.”*

(2) The UMK57 experiment failed. While the authors provide a possible explanation for this failure, it still leaves us without a direct connection between CIN and the observed transcriptomic changes. So it is still impossible to determine which of the observed gene expression changes are due to chromosome imbalance and which are due to CIN. This should be clearly stated in the Discussion.

We agree with the reviewer that we did not clearly mention in the discussion that we were unable to directly investigate the relationship between CIN and the observed transcriptomic changes. We therefore added this to the discussion: *“However, since we were not able to rescue CIN in our clones using UMK57, we can only speculate which transcriptomic changes are driven by CIN and which are caused by chromosome imbalances. Future work aiming to understand the underlying mechanisms of CIN in our clones could open up new strategies to reduce CIN and to directly test this relationship.”*

**Reviewer #3:**

The authors have addressed my previous comments and carried out additional analysis to differentiate mitotic defects based on whether the DNA that does not segregate with the main chromosome mass is CREST+ or acentric. The authors also carried out their proposed research plan. While those results were not conclusive/ positive results, I believe the changes overall strengthen the manuscript and, in my opinion, the manuscript with the current changes is acceptable for publication in PLoS One.

We thank the reviewer for his/her support for publication in PloS One.
